# Convolutional Neural Networks for the Identification of African Lions from Individual Vocalizations

**DOI:** 10.3390/jimaging8040096

**Published:** 2022-04-01

**Authors:** Martino Trapanotto, Loris Nanni, Sheryl Brahnam, Xiang Guo

**Affiliations:** 1 Department of Information Engineering, University of Padua, Via Gradenigo 6, 35131 Padova, Italy; martino.trapanotto@studenti.unibo.it (M.T.); loris.nanni@unipd.it (L.N.); 2 Information Technology and Cybersecurity, Missouri State University, 901 S. National, Springfield, MO 65897, USA; xiangguo@missouristate.edu

**Keywords:** convolutional neural networks, vocal individuality, transfer learning, African lions

## Abstract

The classification of vocal individuality for passive acoustic monitoring (PAM) and census of animals is becoming an increasingly popular area of research. Nearly all studies in this field of inquiry have relied on classic audio representations and classifiers, such as Support Vector Machines (SVMs) trained on spectrograms or Mel-Frequency Cepstral Coefficients (MFCCs). In contrast, most current bioacoustic species classification exploits the power of deep learners and more cutting-edge audio representations. A significant reason for avoiding deep learning in vocal identity classification is the tiny sample size in the collections of labeled individual vocalizations. As is well known, deep learners require large datasets to avoid overfitting. One way to handle small datasets with deep learning methods is to use transfer learning. In this work, we evaluate the performance of three pretrained CNNs (VGG16, ResNet50, and AlexNet) on a small, publicly available lion roar dataset containing approximately 150 samples taken from five male lions. Each of these networks is retrained on eight representations of the samples: MFCCs, spectrogram, and Mel spectrogram, along with several new ones, such as VGGish and stockwell, and those based on the recently proposed LM spectrogram. The performance of these networks, both individually and in ensembles, is analyzed and corroborated using the Equal Error Rate and shown to surpass previous classification attempts on this dataset; the best single network achieved over 95% accuracy and the best ensembles over 98% accuracy. The contributions this study makes to the field of individual vocal classification include demonstrating that it is valuable and possible, with caution, to use transfer learning with single pretrained CNNs on the small datasets available for this problem domain. We also make a contribution to bioacoustics generally by offering a comparison of the performance of many state-of-the-art audio representations, including for the first time the LM spectrogram and stockwell representations. All source code for this study is available on GitHub.

## 1. Introduction

African lions (*Panthera leo*) are famous for their roars, easily heard over vast distances. These long-distance communications serve several functions that range from localizing pride members and coordinating hunting to defining territorial boundaries and avoiding other lions [1,2,3]. Studies have shown that when tensions mount, lions of both genders use roars to judge their chances in a fight [4]. Particularly intimidating is the extreme volume of the confrontational roar.

The characteristic low frequencies of these roars are attributed to the long vocal folds of the larynx shared by all species (except for the snow leopard) of the genus Panthera. A single lion′s roar can last for over a minute and is usually composed of several soft moans followed by a crescendo of full-throated roars that dissolve in a series of short grunts [3]. Both males and females are known to emit these sounds. Studies have examined the attributes of individual roars and have found distinct characteristics in the temporal patterns and acoustic features between males and females [5], but little is known to date about the components that mark individual lions.

For some communication objectives to be realized, a specific lion’s vocalizations must relay reliable information about its identity [6]. The ability of a receiver to decode this information is critical in shaping responses. Lionesses, for instance, by virtue of identifying the roars of alien males encroaching with potential infanticidal intentions [7], can scurry their cubs to safety. Bioacustical studies of other mammals, such as African elephants (*Loxodonta africana*) [7], wolves (*Canis lupus*) [8], and other members of the Panthera genus (e.g., *Panthera tigris*) [9], have revealed features in their calls that broadcast identity; these and other studies also show that this information is transmitted despite the progressive degradation of signals as they propagate through the environment. Thus, it is reasonable to presume the existence of distinctive components in a lion’s roar that robustly express identity.

Lions are not the only mammals who want to identify individual lions by their roars. Biologists are also interested in understanding how lions vocally transmit their identities. Not only can such information further the science of lion bioacoustics, but it can also help conservationists to monitor population densities and investigate species patterns. The study of vocal individuality for passive acoustic monitoring (PAM) and census of wild, urban, and farm animals is becoming an increasingly popular area of research [4,10,11,12,13,14,15,16,17,18,19,20,21].

More and more of these studies are investigating the possibility of using machine learning to identify and monitor individual animals through their vocalizations. In [18], for example, experiments were performed to distinguish thirty-three female Bornean gibbons (*Hylobates muelleri*) from 376 calls recorded in a small region in Sabah, Malaysisa; a Support Vector Machine (SVM) [22] was trained on spectrogram features and Mel-frequency cepstral coefficients (MFCCs) [23]. Using the same dataset, the authors in [19] recently applied an unsupervised learning strategy. In [7], a Hidden Markov model (HMM) was trained on MFCCs for the vocal identification of specific African elephants. In [4], five male lions were classified using a dataset of 164 full-throated roar samples; two classifiers were evaluated on the lion dataset, the k-nearest neighbors (K-NN) algorithm and HMM, both using the fundamental frequency contour sequence as a feature. HMM, often trained on MFCCs, has been applied to identify many individual animals (e.g., tigers [9], songbirds [24], and orangutans [25]) by their unique call signatures. Other classification methods for individual vocal classification include neural networks [26,27] and, in one study, deep learners [12]. In the latter study, a neuro-fuzzy framework that integrated a fuzzy clustering method into a Convolutional Deep Clustering Neural Network (CDCN) was trained to identify individual birds in the wild.

Although deep learning is widely used in bioacoustics (see Section 2), the main problem applying deep learners to individual vocal classification is that they need to be trained on large datasets to avoid overfitting. Despite some large-scale accumulations of species vocalizations (for example, the dataset for North Atlantic right whale upcalls [28] and the Bird Audio Detection challenge [29], both containing samples numbering in the tens of thousands), a scarcity of labeled training data has been a significant impediment for many bioacoustic classification tasks. This dearth of data is even more pronounced when it comes to individual animal classification due to the compounded difficulties and expenses involved in collecting and labeling individual animal calls.

Some powerful machine learning techniques for handling small sample sizes in bioacoustic problems generally include data augmentation [29,30,31], triplet loss [32], and transfer learning [33]. Some of the strongest results in bioacoustic studies (see [29,31,34]) have been produced with deep learners using data augmentation, a technique that adds variation and noise to the data to generate new samples. Good results have also been obtained for whale identification using transfer learning [34]. Transfer learning is a technique that takes a pretrained network (often trained on datasets numbering in the millions) and replaces the last fully connected layer of the deep learner with a new one that is explicitly trained on a set of samples representing a different task.

The objective of this work is to apply state-of-the-art deep learning methods to the problem of identifying individual lions from their roars. Eight audio representations (described in Section 3.3) are considered: classic MFCC, spectrogram, Mel spectrogram [23], and several new representations, including VGGish [35], stockwell [36], and those based on the recently proposed LM spectrogram [37]. We evaluate our approach on the small dataset used in [4] (detailed in Section 4) and handle the problem of overfitting using two methods. The first method is transfer learning: three CNNs (VGG16, ResNet50, and AlexNet) pretrained on ImageNet (briefly described in Section 3.2) are retrained on each of the audio representations. The single models and their combinations are evaluated and compared as reported in Section 5. The second method for handling the small size of the lion dataset was to divide it using two different Leave One Out Cross-Validation (LOOCV) strategies; one dataset was used for training and testing, and the other for evaluating the best ensembles. Our proposed system significantly improves the classification rate from 91.5% in [4] to over 98% accuracy, with our results corroborated using the Equal Error Rate (EER).

In this study, we make several contributions to the field of individual vocal classification:

- We demonstrate not only that it is possible, with caution, to use transfer learning with single pretrained CNNs on the small datasets available for this problem domain, but that it is also valuable to build ensembles of these deep learners.
- We compare the performance of many state-of-the-art audio representations. Additionally, we also evaluate the LM spectrogram and stockwell for the first time, to the best of our knowledge, on a bioacoustic problem.

Finally, because we propose a more realistic testing protocol for the lion dataset with results calculated using EER and an ad hoc testing protocol (a standard indicator for validating the discrimination capabilities of biometric systems), a comparison of our results with future works on this dataset will be better validated.

## 2. Related Work in Bioacoustic Classification

### 2.1. Convolutional Neural Networks (CNNs)

In the last decade, the prevailing machine learning paradigm has been deep learning, a method that automatically discovers relevant features for the classification task at hand. Deep learners are multilayer networks that learn representations of data through the hierarchical composition of modules that transform features into progressively higher levels of abstraction. One of the most powerful deep learners for image classification is CNN. Although it was first developed in the 1980s [38], interest in CNN snowballed after it achieved outstanding results in the ImageNet Large Scale Visual Recognition Challenge, where a GPU-based CNN won the competition [39]. CNNs are composed of convolutional layers that apply a series of filters to the input data, generating different output vectors for each filter along with a single weight. Interspersed at intervals are pooling layers. The CNN architecture results in networks that are faster, more reliable, and more effective in image recognition tasks [39,40,41,42].

Because audio representations are typically based today on visual representations of sound (see Section 2.2), deep learning has been applied to many bioacoustic problems, and the number of deep learning studies in this area is accelerating. Some samples of studies published in this field in the last couple of years include deep learning systems for whale detection, where a Multimodel Deep Learning algorithm was developed in [43], a ResNet in [28], and a CNN-based approach combined with Long Short-Term Memory in [34]. Two deep CNNs (a linear and a pyramidal) were trained for manatee identification in [44], a tiny CNN for bat echolocation calls was developed in [45], and a CNN to detect and classify bearded seal vocalizations in [46]. For bird species identification, two recent examples include [47], where CNNs were trained on an owl dataset, and [48], where a ResNet architecture and VGG16 were trained and compared on the Cornell Bird Challenge Dataset.

### 2.2. Visual Audio Representations

The first and one of the most popular audio representations in bioacoustics is the spectrogram, also known as the sonograph. Spectrograms are colored representations of the signal frequency spectrum (y-axis) versus time (x-axis). The brighter the color in a frequency range, the more concentrated the sound in that range; completely dark areas represent missing frequencies. A small sample of bioacoustic articles classifying spectrograms with CNNs published the last two years includes [12,47] for bird species identification, [33] for fish classification, [49,50,51] for whale detection, and [52] for comparing context-dependent call sequences in bats.

The linear approach offered by spectrograms fails to represent specific sound characteristics and patterns [53]. An alternative is the Mel-spectrogram [23], which is a signal representation derived from the spectrogram that substitutes the normal frequency scale with the nonlinear Mel scale, taken from 20th-century psychoacoustics studies that explored the relationship between frequency and human perceived pitch changes [54]. Similar to the Mel spectrogram is the Mel-frequency cepstrum (MFC) [23], an acoustic representation based on a linear cosine transform of a log power spectrum on the Mel scale. The difference between the Mel-spectrum and MFC is that the frequency bands of MFC are equally spaced, whereas the Mel-spectrum is based on the way the human ear affects the sound, which may or may not be appropriate for modeling how animals identify each other from their vocalizations. The Mel-frequency coefficients that form MFC are known as MFCC [23], yet another audio representation. MFCC coefficients were introduced in the 1980s and remain a fundamental representation in speech recognition and machine learning for audio [55]. Some recent studies in bioacoustic classification based on the Mel scale include [56] for modeling cattle vocalizations, [57,58,59,60] for automatic bird voice classification, [61,62] for whale call classification, [63] for recognizing ultrasonic mice vocalizations, [64] for automatically monitoring environmental sounds, such as birds, carcadia, field crickets, and background noise, [65] for automatic annotation of marmoset calls, and [66,67,68] for identifying toads, frogs, and other wildlife.

Finally, a representation relevant to this paper that has recently been applied to bioacoustic classification is VGGish [35], which refers to the extraction of feature vectors generated by a pretrained VGG network. This representation has recently been applied to birdsong parsing [69] and whale classification [70] with great success.

## 3. Materials and Methods

### 3.1. Overview of the System

**Figure 1:**
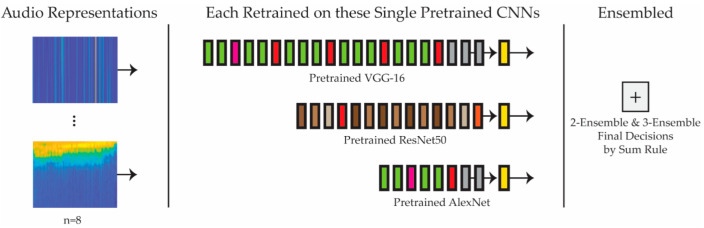
Overview of the proposed system. Each audio representation is trained on each of the three pretrained CNNs, some of which are ensembled (in groups of two or three) by Sum Rule.

Figure 1 provides a schematic of the system proposed in this study. Eight audio representations are fed into three separate CNNs (VGG-16, ResNet50, and AlexNet), pretrained on ImageNet. The results of these single networks trained on each of the representations are then evaluated. Once this evaluation is completed, various ensembles that combine, using the sum rule, two or three of the best single models are then compared with and evaluated against each other. For a very recent paper validating image quality assessment with CNNs and decision fusion, see [71].

### 3.2. Pretrained CNNs

As already noted, the models evaluated in this study are AlexNet, VGG-16, and ResNet-50. These famous CNN models are briefly described below.

#### 3.2.1. AlexNet

AlexNet is one of the most influential and famous CNNs of the last decade, winning the ImageNet Challenge in 2012 by a wide margin and propelling the whole technology into the forefront overnight. This network combines convolutional and max-pooling layers with the ReLU activation function. GPU-based training with CUDAs can easily and significantly cut down the training time on AlexNet, with its 60 million parameters [39].

#### 3.2.2. VGG-16

VGG-16 is another CNN model that was developed by Oxford University for the 2014 ImageNet challenge. It is an evolution of AlexNet. In the manner of AlexNet, VGG-16 has similar convolutional and max-pooling layers combined with a final fully connected classification layer [42]. It differs from AlexNet in its sheer size and complexity: VGG-16 has more than 138 million parameters, due to its smaller filter size (down to 3 × 3) and deeper structure with 16 layers. This jump in complexity negatively affects the performance, making this network heavier on resources for training, testing, and storage. These added costs reflect the concomitant advances in performance: VGG is still considered a state-of-the-art CNN [42].

#### 3.2.3. ResNet-50

ResNet is a newer design that attempts to fix issues observed with deeper models [72]. Even though CNN technology has been popular for a relatively short time, some questionable maxims have become mainstream, perhaps the most touted being “deeper is better”. However, it is not that uncommon for very deep models to stop or even reverse performance gains, a circumstance linked to multiple factors, including the vanishing gradients in backpropagation with deep layers (see [73] for a discussion).

The ResNet innovation was the Residual Block, which implements a “short-cut” between layers, allowing lower-level features to reach deeper in the network unscathed and permitting the network to more easily learn identity functions. These skip connections, as they are called, allow the networks to reach 161 layers while still maintaining performance gains. This design is easier to train than the VGG model and requires less computing time [73].

### 3.3. Audio Representations

**Figure 2:**
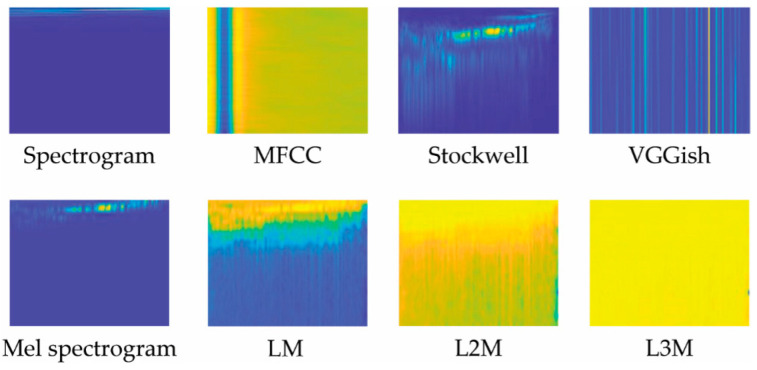
Illustration of the eight audio representations used in this work, each generated from our dataset’s first sample.

**Figure 3:**
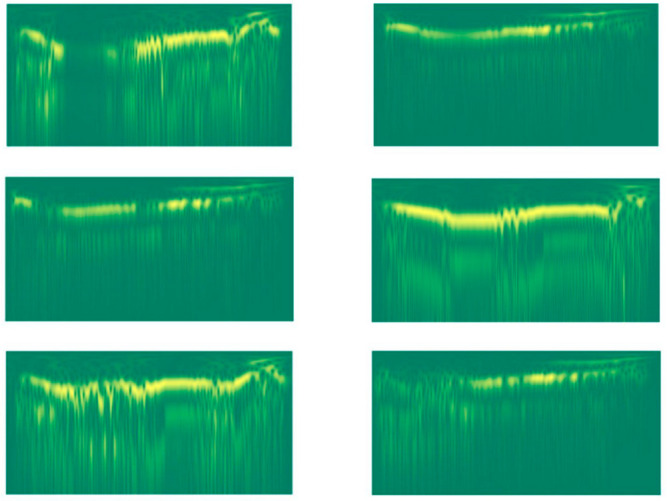
Stockwell images of two different lions (the three images of each column are related to a given lion).

Eight audio representations were evaluated in this work. As noted in Section 2, two are staples in bioacoustic classification: MFCC and Spectrograms. VGGish has only recently been explored in this task domain. Stockwell and our variations of LM are examined in this paper for the first time. These representations are illustrated in Figure 2 and Figure 3 and are detailed below.

#### 3.3.1. Spectrogram

Spectrograms show how the frequency content of a signal varies in time by displaying the power distribution in the signal’s frequency domain for each time slice; this is in contrast to how the Fourier Transform collapses the whole signal into a single frequency distribution, ignoring the time dimension [74].

This representation is generated using the Short-Time Fourier Transform (STFT), calculated by extracting the Fourier Transform of the convolution between the original signal and a window for extracting a single time slice:

(1) $$\mathrm{STFT}\{x(t)\}(\tau ,\;\omega )=\int_{-\infty }^{\infty }x\left(\tau \;\right)w\left(t-\tau \;\right)e^{-i\omega t}dt,$$

where w(t) is the window signal, typically a Gaussian or Hammon function [75]; x(τ)  is the signal undergoing transformation; *τ* is the time axis; and ω is the frequency axis.

#### 3.3.2. Mel Spectrogram

A Mel spectrogram is a classical spectrogram in which the frequencies are converted to the Mel scale. The standard formula for the Mel scale [23] is

(2) $$m=2595\log_{10}\left(1+f/700\right),$$

where f hertz is the frequency.

Because the resulting values from this implementation are distributed on a small interval around 0, too small to be recognized by a neural network, we applied multiple normalization techniques, such as linearly normalizing in the range [0–1000] or taking the natural log (see Section 5 for more details on scaling operations).

#### 3.3.3. LM, L2M, L3M

These features, derived from the Mel Spectrogram, were recently developed in [37] and provide an approach based on the log10 of the Mel Spectrogram. This approach highlights the most relevant signal components. The algorithm can be iterated beyond the log10 function, known as LM, to define the L2M and L3M features:

(3) $$\mathrm{LM}=\frac{\left[10\times \log_{10}\left(S\right)-10\times \log_{10}\left(ref\right)+60\right]}{10}$$

(4) $$L2M=\frac{\left[10\times \log_{10}\left(\mathrm{LM}\right)-10\times \log_{10}\left(ref\right)+10\right]}{40}\;$$

(5) $$L3M=\frac{\left[10\times \log_{10}\left(L2M\right)-10\times \log_{10}\left(ref\right)+60\right]}{10}\;$$

where S is the Mel Spectrogram, and ref are the reference values, defined as the max values of the signal [37].

The implementation in [37] has some limitations, however. LM produces negative values, which in the logarithm in ℛ  are not defined.

To work around this difficulty, we redesigned the algorithm as follows:

LM = 100 × log_10_(*S*) (6)

L2M = 100 × log10(LM − *minref*) (7)

L3M = 100 × log10(L2M − *minref*) (8)

where S is the Mel Spectrogram, as in Equations (3)–(5), and minref are the minimum values of the signal. The subtraction of minref from the features generated in previous iterations (LM and L2M) shifts all values above 0.

#### 3.3.4. MFCC

MFCC [23] is generated by first calculating the Short-Time Fourier Transform of the signal, applying it to the Mel Bank Filters to obtain the Mel spectrogram, taking the log of this spectrum, and finally applying an inverse Fourier transform on the result.

This process can be repeated for windowed segments of the signal, in a fashion similar to the way the spectrogram was generated, to obtain the MFCC as the signal evolves over time:

(9) $$\mathrm{MFCC}={\left|F^{-1}\left(\log\left(mel\left({\left|F\left(\mathrm{Sig}\right)\right|}^{2}\right)\right)\right)\right|}^{2}$$

where F is the Fourier transform, *mel* is the conversion to the *mel* scale, and  Sig is the input signal.

Conceptually, the Mel bank passage shapes the signal to represent human frequency perception, while the power log passage represents human sound perception. The inverse Fourier transform serves to make these coefficients less correlated [76].

#### 3.3.5. Stockwell

The Stockwell transform [36], also known as the S Transform, is a derivation of the STFT that is similar to the Gabor Transform. Stockwell is the Fourier transform of a signal convoluted with a modified Gaussian function

This transform is quite computationally expensive. As a result, we had to decimate the input signal by a factor of 10 to lower the computational time required to generate the features. Examples of the resulting stockwell images are shown in Figure 3.

#### 3.3.6. VGGish Features

VGGish features (treated as an image) represent an audio file by extracting features from the last fully connected layer of a VGG network pretrained on audio data. VGGish features are a 128-element feature vector over time. This study used the default setting in the MATLAB VGGish model (see https://it.mathworks.com/help/audio/ref/vggish.html accessed on 21 February 2022). The model was trained by Google in 2017 on audio taken from a large YouTube dataset [35].

## 4. Data Collection and Cross-Validation Techniques

### 4.1. Data Collection

The dataset used in this work was obtained from the study reported in [4], conducted in the Bubye Valley Conservancy, a private reserve in southern Zimbabwe, approximately 3400 km^2^. This conservancy is home to many African megafauna species.

Lions are primarily active at night, as are their vocalization activities; this makes associating the identity of an animal with a collected sample challenging. A novel approach was taken in [4] to match lions with their roars. Five males and three females were narcotized, captured, and fitted with a bio-logger system composed of an accelerometer, microphone, and magnetometer encased in epoxy housing. Once the animals were released, the sensors collected between four and ten days of data before the batteries failed. The audio files were encoded with a bit depth and sampling rate of 8-bit/16 kHz. When the animals were recaptured, the data were downloaded, and the recordings were manually processed to isolate the roars by inspecting the spectrograms and tagging the samples. A tracked animal was judged to be the one roaring by examining the volume (it was assumed that the tracked lion’s roar would be noticeably louder than others) and by inspecting the accelerometer data (roaring lions have a distinct head movement pattern). Any noise or interference, including anthropogenic noise or coinciding roars, was grounds for removing a sample from the dataset. The crescendoing roars, which come in bouts, were split up such that only the first three were included in the dataset [4]. The resulting dataset was composed of 164 full-throated roar samples divided across five males. No female roared during the recording period.

### 4.2. Cross-Validation Dataset Design

Because the number of samples in the lion roar dataset is small, the Leave One Out Cross-Validation (LOOCV) paradigm was used to construct two different datasets, labeled as follows:

1. ERR Day: each test set is the full-throated samples collected on a single day, with all the remaining making up the training set. The Day dataset is thus a 20-fold dataset.
2. ERR Bout: each test set is a single bout of roars, between 1 and 3 samples, with the rest becoming the training set. Bout is thus a larger set, with around 74 folds.

We decided not to separate the single samples as test sets to avoid the strong correlation that roars from the same bout are expected to have; separating single samples could compromise the independence of the test and training sets.

Most of the training and testing were performed on the Day set because of its smaller size, which reduced the computational time. The larger Bout set was reserved for performance testing.

### 4.3. Equal Error Rate (EER) Dataset Design

To validate the performances of our model further, we calculated the Equal Error Rate (EER), as this is a standard technique for validating the discrimination capabilities of a biometric system. This value is calculated by finding the threshold value along the Receiver Operating Characteristic (ROC) curve where the False Acceptance Rate and the False Rejection Rate are equal. Generally, a lower ERR indicates more accuracy.

To calculate EER, we needed to restructure the cross-validation dataset into a binary problem, i.e., into a “one-to-many” design. For each fold, a lion was selected, and its roars were tagged as class 1. All other lions were assigned to class 2. This approach also enables this system to recognize animals from outside the dataset. We kept the Leave One Out technique, retaining a single sample from each class in Day or Bout for testing and all that remained for training. This procedure produced two EER datasets, an “EER Day” and an “EER Bout” dataset. All networks and their combinations were trained on both dataset versions to validate the results.

## 5. Experimental Results

In the experiments that follow, each of the audio representations was fed into a separate CNN model. As described in Section 4, all tests were performed on the Day Cross-Validation set, consisting of twenty different training-test divisions of the same dataset. In contrast, the Bout dataset was kept only to evaluate the results, specifically for the ten best-performing setups to confirm the rankings.

Some audio representations had to be manipulated more than others. As noted in Section 3.3.2, the Mel Spectrogram, for example, needed to be heavily modified once processed with the standard MATLAB library, as the network could not converge due to the small data range between 0 and 0.025. Simple normalization techniques gave much clearer results, and more complex log-based procedures pushed our interests toward L2M and L3M features. Other representations, such as the MFCC and VGGish features, were kept as simple as possible due to their specific nature and the networks’ abilities to converge on the representation input. In the end, we obtained satisfactory results with the best performing network, a VGG16 LM system (95.6% accuracy on the Day dataset).

In Table 1, Table 2, Table 3, Table 4 and Table 5, the word spectrogram is abbreviated S to reduce table clutter, and adjustments to the representation patterns, aside from those mentioned in Section 3.3, are abbreviated and explained as follows:

- Min-max: the pattern is scaled in the range of [0–255] with this formula: pattern=(pattern−min_p)/(max_p−min_p)∗255;
- db: the formula for this adjustment is pattern=10∗log10(pattern+1e−16).
- box_n (box normalize): same as min-max, except that the pattern is scaled in the range [0–Constant]: pattern=(pattern−min_p)/(max_p−min_p)∗Constant.

Please refer to the GitHub MATLAB code for implementation details (see the Data Availability Statement at the end of this paper).

The results in Table 1 show the best ten single networks. The results on the Bout dataset might be partially due to over-fitting; the small test set, often just one sample per fold, also had an effect. While these results cannot accurately measure a system’s performance, they lend weight to the more realistic results from the Day dataset.

As described before, our ensemble results were calculated by recording the classification scores of each run and combining them to find the best combinations. This improved performance significantly, obtaining for a two-ensemble (VGG16 MFCC + VGG16 LM) the best performance of 97.6% and for a three-ensemble (VGG16 S + AlexNet LM + VGG16 LM) a maximum of 98.6%. It is noteworthy that each of these performances is shared by different ensemble pairs: 97% for the two-ensemble and 98% for each of the three-ensemble, as shown in Table 2 and Table 3.

### EER Dataset Validation

The EER results were used to validate the ability of the networks to discriminate the samples accurately, as described in Section 4.3. The results in Table 4, Table 5 and Table 6 show the EER results for the best performing networks for both the Day and Bout datasets. The EER results corroborate our previous analysis, showing how these networks are capable of one-to-many distinctions.

In Table 7, we report the performance of three other CNNs combined with LM S and the ERR Bout testing protocol. As can be observed, the performance is similar to the other networks

In Table 8 and Table 9, we report some computation time results. In Table 8, we report the computation test time for classifying a batch of 100 spectrograms and, in Table 9, the computation times for representing an audio file as a matrix. The computation times were obtained using a Titan XP-i7-6900K, 3.2 GHz, with 64 GB Ram, and MATLAB 2021.

## 6. Discussion

We can see the high-performance results for many of our representation-network combinations: over 90% for almost all the top ten performers alone and exceeding 95% for all the ten best two-ensembles. It should be noted how prominent the LM feature is in all three tables, especially in combination with the VGG16 network, which is the most common combination in all our results. Another prominent feature is the original spectrogram and some of its variations, and MFCC demonstrates great performance on its own and in ensembles. These results significantly exceed the 91% accuracy of the Hidden Markov Model used in [4] and further illustrate the power of using CNNs on audio tasks, especially in novel studies such as this one.

While the results are encouraging, we need to keep in mind that this dataset was not only small but also very limited in variety. We only had 164 audio samples collected from five animals. We handled the small data size using transfer learning and the division of our datasets into two separate leave one out datasets: data augmentation was not explored, and no noise was added. The ensemble results were generated by combining all possible results and selecting the best ones.

The reported performance on the lion dataset, however, clearly shows that deep learning strongly outperforms previous handcrafted approaches as tested in the lion roar classification problem. More research is needed to fully assess the performance in other bioacoustic problems on the methods proposed in this paper and the value of using the LM spectrogram and stockwell representations.

## Figures and Tables

**Table 1:** Performance Accuracy of the Ten Best Single Networks on the ERR Day and ERR Bout Datasets.

Network and Feature	Day	Bout
VGG16 LM S	95.67%	98.9%
VGG16 dB LM S	94.50%	100%
AlexNet custom LM S	93.66%	96.8%
ResNet50 S	91.97%	97.6%
VGG16 S	91.10%	97.2%
ResNet50 min-max S	90.59%	95.9%
AlexNet box_n Mel S	90.17%	96.3%
VGG16 MFCC	90.10%	94.8%
VGG16 L2M S	89.78%	94.6%
VGG16 min-max S	89.69%	97.6%

**Table 2:** Performance Accuracy of the 2-Ensemble on the ERR Day and ERR Bout Datasets (sorted by performance).

Network 1	Network 2	Day	Bout
VGG16 MFCC	VGG16 LM S	97.64%	99.3%
ResNet50 S	AlexNet LM S	97.61%	98.7%
ResNet50 S	VGG16 LM S	97.61%	99.4%
ResNet50 S	VGG16 dB LM S	97.45%	99.4%
ResNet50 min–max S	VGG16 LM S	97.31%	100%
AlexNet min–max scaled S	VGG16 LM S	97.06%	100%
ResNet50 min–max scaled S	VGG16 LM S	96.90%	99.1%
ResNet50 Mel S	VGG16 LM S	96.78%	99.5%
AlexNet dB LM S	VGG16 LM S	96.78%	99.5%
VGG16 min-max scaled S	VGG16 LM S	96.68%	97.6%

**Table 3:** Performance Accuracy of 3-Ensemble on the ERR Day and ERR Bout Datasets (sorted by performance).

Network 1	Network 2	Network 3	Day	Bout
VGG16 min–max S	AlexNet LM S	VGG16 LM S	98.67%	100%
ResNet50 S	ResNet50 Mel S	VGG16 dB LM S	98.42%	100%
ResNet50 min–max S	AlexNet LM S	VGG16 dB LM S	98.42%	100%
ResNet50 S	VGG16 MFCC	VGG16 LM S	98.42%	100%
ResNet50 S	VGG16 dB LM S	VGG16 LM S	98.19%	100%
AlexNet min–max Mel S	VGG16 dB LM S	VGG16 LM S	98.17%	100%
VGG16 dB LM S	AlexNet box_n Mel S	VGG16 LM S	98.17%	100%
ResNet50 S	AlexNet VGGish	VGG16 dB LM S	98.14%	100%
ResNet50 S	AlexNet LM S	VGG16 LM S	98.14%	100%
ResNet50 min–max S	AlexNet dB LM S	VGG16 LM S	98.14%	100%

**Table 4:** Equal Error Rate (EER) of Best Performing Single Network on the EER Day and EER Bout Datasets (sorted by performance).

Networks and Feature	Day	Bout
VGG16 LM S	5.62	0.53
VGG16 dB LM S	3.67	0.69
AlexNet LM S	4.87	2.38
ResNet50 S	6.97	1.92
VGG16 S	7.27	2.38
ResNet50 min–max S	7.87	1.84
AlexNet box_n Mel S	3.82	2.99
VGG16 MFCC	11.6	6.07
VGG16 L2M Mel S	9.82	6.07
VGG16 min-max S	7.49	2.45

**Table 5:** Equal Error Rate (EER) of Best Performing 2-Ensemble on the EER Day and EER Bout Datasets (sorted by performance).

Network 1	Network 2	Day	Bout
VGG16 MFCC	VGG16 LM S	6.82	1.53
ResNet50 S	AlexNet LM S	2.69	0.53
ResNet50 S	VGG16 LM S	5.02	0.53
ResNet50 S	VGG16 dB LM S	4.34	0
ResNet50 min–max S	VGG16 LM S	2.47	0.53
AlexNet min–max S	VGG16 LM S	5.47	0.53
ResNet50 min–max S	VGG16 LM S	4.20	0.53
ResNet50 Mel S	VGG16 LM S	4.49	0.53
AlexNet dB LM S	VGG16 LM S	6.07	1.15
VGG16 min–max S	VGG16 LM S	6.75	0.69

**Table 6:** Equal Error Rate (EER) of Best Performing 3-Ensemble on the EER Day and EER Bout Datasets (sorted by performance).

Networks	Networks	Networks	Day	Bout
VGG16 min–max S	AlexNet c. LM S	VGG16 LM S	3.00	0.53
ResNet50 S	ResNet50 Mel S	VGG16 dB LM S	5.09	0
ResNet50 min–max S	AlexNet LM S	VGG16 LM S	3.00	0.53
ResNet50 S	VGG16 MFCC	VGG16 LM S	5.47	0.61
ResNet50 S	VGG16 dB LM S	VGG16 LM S	3.89	0.07
AlexNet min–max Mel S	VGG16 dB LM S	VGG16 LM S	3.60	0.53
VGG16 dB LM S	AlexNet box_n Mel S	VGG16 LM S	2.40	0.15
ResNet50 S	AlexNet VGGish	VGG16 dB LM S	6.22	0.07
ResNet50 S	AlexNet LM S	VGG16 LM S	2.47	0.23
ResNet50 min–max S	AlexNet dB LM S	VGG16 LM S	3.29	0.53

**Table 7:** Performance of other CNN topologies.

Network and Feature	ERR Bout
VGG19 LM S	98.9%
ResNet101 LM S	97.6%
MobileNetV2 LM S	96.3%

**Table 8:** Classification time (seconds) for a batch of 100 spectrograms.

Networks	Classification Time
AlexNet	0.148
ResNet50	0.299
VGG16	0.688

**Table 9:** Computation times (seconds) for representing audio file as an image.

Networks	Computation Time
Spectrograms	0.015
MFCC	0.009
Stockwell	0.340
VGGish	0.015
Mel spectrogram	0.055

## Data Availability

The data that support the findings of this study are available from the original author of the lion dataset: Matthew Wijers. The MATLAB code for all the data augmentation methods is available at https://github.com/LorisNanni (accessed on 24 February 2022).

## List of Acronyms

**Acronym**	**Full Term**
CDCN	Convolutional Deep Clustering Neural Network
CNN	Convolutional Neural Network
EER	Equal Error Rate
HMM	Hidden Markov model
K-NN	K-Nearest Neighbors
LOOCV	Leave One Out Cross-Validation
MFC	Mel-frequency cepstrum
MFCCs	Mel-Frequency Cepstral Coefficients
PAM	Passive Acoustic Monitoring
ROC	Receiver Operating Characteristic
SVM	Support Vector Machines
**Acronym**	**Explanation**
Bout Dataset	Each test set is a single bout of roars, between 1 and 3 samples (see Section 4.1)
box_n	Adjustment to a representation: pattern=(pattern−min_p)/(max_p−min_p)∗Constant
Day Dataset	Each test set is the full-throated samples collected on a single day (see Section 4.1)
ERR Day	Equal Error Rate Day dataset design (see Section 4.3)
ERR Bout	Equal Error Rate Bout dataset design (see Section 4.3)
db	Adjustment to a representation: pattern=10∗log10(pattern+1e−16)
LM and L2M	Features derived from the Mel Spectrogram (see Section 3.3.3)
Min-max	Adjustment to a representation: (pattern=(pattern−min_p)/(max_p−min_p)∗255)
S	Spectrogram
